# Persistent Organic Pollutants and the Association with Maternal and Infant Thyroid Homeostasis: A Multipollutant Assessment

**DOI:** 10.1289/EHP152

**Published:** 2016-05-24

**Authors:** Vivian Berg, Therese Haugdahl Nøst, Rolf Dagfinn Pettersen, Solrunn Hansen, Anna-Sofia Veyhe, Rolf Jorde, Jon Øyvind Odland, Torkjel Manning Sandanger

**Affiliations:** 1 Diagnostic Clinic, University Hospital of North Norway, Tromsø, Norway; 2 NILU–Norwegian Institute of Air Research, Fram Centre, Tromsø, Norway; 3 Department of Community Medicine, UIT–the Arctic University of Norway, Tromsø, Norway; 4 Norwegian National Unit for Newborn Screening, Women and Children’s Division, Oslo University Hospital, Oslo, Norway; 5 Institute of Clinical Medicine, UIT–the Arctic University of Norway, Tromsø, Norway; 6 Department of Public Health, University of Pretoria, Pretoria, South Africa

## Abstract

**Background:**

Disruption of thyroid homeostasis has been indicated in human studies targeting effects of persistent organic pollutants (POPs). Influence on the maternal thyroid system by POPs is of special interest during pregnancy because such effects could impair infant thyroid homeostasis.

**Objectives:**

We investigated the association between POPs and thyroid-stimulating hormone (TSH) and thyroid hormones (THs) in mother and child pairs from the Northern Norway Mother-and-Child Contaminant Cohort Study (MISA).

**Methods:**

Nineteen POPs and 10 thyroid parameters were analyzed in serum from 391 pregnant women in their second trimester. In addition, TSH concentrations in heel-prick samples from the infants were analyzed by the Norwegian Newborn Screening program. Association studies with a multipollutant approach were performed using multivariate analyses; partial least squares (PLS) regression, hierarchical clustering, and principal component analysis (PCA).

**Results:**

Several POPs were significantly associated with TSH and THs: a) PFOS was positively associated with TSH; b) PCBs, HCB, and nonachlors were inversely associated with T3, T4, and FT4; and, c) PFDA and PFUnDA were inversely associated with T3 and FT3. After mutual adjustments for the other contaminants, only PFDA and PFUnDA remained significantly associated with T3 and FT3, respectively. Infants born to mothers within the highest TSH quartile had 10% higher mean concentrations of TSH compared with children born to mothers in the lowest TSH quartile.

**Conclusion:**

The present results suggest that background exposures to POPs can alter maternal thyroid homeostasis. This research contributes to the understanding of multipollutant exposures using multivariate statistical approaches and highlights the complexity of investigating environmental concentrations and mixtures in regard to maternal and infant thyroid function.

**Citation:**

Berg V, Nøst TH, Pettersen RD, Hansen S, Veyhe AS, Jorde R, Odland JØ, Sandanger TM. 2017. Persistent organic pollutants and the association with maternal and infant thyroid homeostasis: a multipollutant assessment. Environ Health Perspect 125:127–133; http://dx.doi.org/10.1289/EHP152

## Introduction

Human endocrine systems such as the thyroid are susceptible to disruption by naturally occurring and human-made compounds, possibly by affecting the hormone homeostasis. Endocrine-disrupting abilities have been suggested for persistent organic pollutants (POPs), of which two major groups are perfluoroalkyl substances (PFASs) and organochlorines (OCs). POPs are persistent substances that have been emitted to the environment (Lohmann et al. 2007; Prevedouros et al. 2006). Still, PFASs and OCs have different chemical properties and histories of production and use. Diet is suspected to be the major current exposure pathway to POPs for humans (Malisch and Kotz 2014; Vestergren and Cousins 2009). In addition, PFASs are passed to humans through air, house dust, drinking water, and water-based beverages (Eschauzier et al. 2013; Haug et al. 2011; Ullah et al. 2011).

Disruption of thyroid homeostasis following POP exposure has been observed in animal experiments and indicated in human studies (Boas et al. 2012). Influence on the maternal thyroid system by POPs is of special interest during pregnancy because such effects could delay and impair fetal and neonatal development (Massart and Meucci 2007). The thyroid endocrine system is critical for regulating energy homeostasis, metabolic pathways, and the growth and differentiation of many tissues and organs. Thyroid-stimulating hormone (TSH) regulates the production of the thyroid hormones (THs), triiodothyronine (T_3_), and thyroxine (T_4_). Maternal T_4_ is the sole source of TH to the developing fetal brain before the onset of the fetal thyroid function at approximately 20 weeks gestation (Morreale de Escobar et al. 2004). The fetus is still dependent on maternal THs throughout the gestational period, and inadequate transfer of these may alter thyroid homeostasis in infants also the first weeks after birth (Blackburn 2013).

The major metabolic processes (e.g., metabolism of fat, glucose, protein, and micronutrients) increase during the pregnancy along with an expansion of blood volume, to meet the demand of uterus and fetal development. During the first two trimesters of pregnancy, there are marked changes in the maternal hypothalamic–pituitary–thyroid (HTP) axis to increase the availability of THs in blood. In short, these changes lead to a 2- to 3-fold increase in thyroid hormone–binding proteins (TH-BPs), and a subsequent decrease in levels of free thyroxine (FT_4_) and free triiodothyronine (FT_3_) followed by an increased production of T_3_ and T_4_. Changes in individual TH levels throughout pregnancy vary by gestational age, number of fetuses, and study population, but generally, the woman achieves a new steady state in HTP function at the end of the second trimester that is maintained until delivery (Blackburn 2013). Pregnancy-induced changes in thyroid physiology affect laboratory interpretation, and presently there are no universally accepted reference ranges (Fitzpatrick and Russell 2010).

The potential influence on thyroid homeostasis by POPs in background exposed populations are of interest, especially if POPs can mimic or inhibit the response of natural hormones even at low doses (Vandenberg et al. 2012). Linear effects of many hormones exist up to a dose that occupies about 10% of receptors; at higher doses, occupancy rate does not linearly increase as the dose of the hormone increases (Welshons et al. 2003). Similarly, some POPs might be more potent endocrine disruptors at low concentrations (Ruzzin et al. 2010). Therefore, the present study aimed to perform a multipollutant assessment of the effect of background exposures of POPs on thyroid homeostasis in mother–child pairs in Northern Norway.

## Materials and Methods

### Study Participants and Collection of Blood Samples

The selected subjects in the present study were 391 mother–child pairs from the Northern Norway Mother-and-Child Contaminant Cohort Study (MISA), which consists of 515 enrolled pregnant women recruited from May 2007 to June 2009. The 391 participants with complete data sets consisting of maternal serum concentrations of PFASs, OCs, thyroid parameters, and infant TSH concentrations were initially included in the study. Women with self-reported thyroid-related disease (*n* = 16) and/or twin pregnancy (*n* = 6) were excluded and 370 mother–child pairs were included in the statistical analyses. The mothers answered a detailed questionnaire about diet and lifestyle at enrollment, and donated a blood sample during their second trimester (median, gestational week 18). The women were requested to fast or eat a light, nonfatty breakfast no later than 2 hr before the blood sampling. Blood samples of infants were collected 3 days after birth. Detailed information about the study group characteristics, ethics approvals, the food frequency questionnaire (FFQ), and the blood collection procedures have been reported elsewhere (Hansen et al. 2010; Veyhe et al. 2012). Planning of the project took place in 2006, and approvals were obtained from the Regional Committee for Medical and Health Research Ethics and the Norwegian Data Inspectorate.

## Chemical Analyses

### PFAS Analyses

Blood samples were analyzed for a broad selection of PFASs. A total of 26 PFASs were initially screened for in a subgroup of 50 serum samples. Compounds detected above the limit of detection (LOD) in > 20% of the samples were further quantified in all serum samples. Detailed information about the compounds, sample preparation, extraction method, analytical method, reagents, and instrumentation has been reported elsewhere (Berg et al. 2014; Hanssen et al. 2013). Briefly, PFASs were determined in serum samples using sonication-facilitated liquid–liquid extraction, activated ENVI-Carb™ (Sigma Aldrich) cleanup (Powley et al. 2005) and analyzed by ultrahigh-pressure liquid chromatography triple–quadrupole mass-spectrometry (UHPLC-MS/MS).

### OC Analyses

The methods employed for the OC analyses have been described in detail in Hansen et al. (2010). Briefly, internal standards, formic acid, and deionized water were added to 2 mL serum sample and left in the refrigerator overnight before being extracted through an HLB solid-phase extraction (SPE) column using dichloromethane. Further cleanup involved elution of compounds from Florisil columns with n-hexane/dichloromethane. OCs were identified and quantified in the extracts with a gas chromatograph/mass spectrometer operated in electron impact mode. Assessment of isotopic mass ratios, blank samples, and standard reference materials ensured the quality of the results. Finally, lipids were determined enzymatically, and the summed amount of lipids was calculated as described by Akins et al. (1989).

The quality of the PFAS and OC analyses was assured through repetitive analysis of blank samples and reference samples. Additionally, our laboratory participates in the international Artic Monitoring and Assessment Programme ring test for POPs in human serum (Institut national de santé publique du Québec 2014). Interlaboratory comparisons and reference samples indicate that the uncertainties of our analyses are within ± 15–20% of the assigned values. Further details on quality control issues have been published elsewhere (Berg et al. 2014; Hansen et al. 2010). The linear perfluorooctane sulfonate (PFOS) isomers were chromatographically separated from the branched isomers and quantified separately. Summed concentrations of isomers were used in the statistical analyses.

### TH and TH-BP Analyses

Determination of maternal THs, TH-BPs [thyroxine-binding globulin (TBG), transthyretin (TTR) and albumin], thyroxine-binding capacity, and anti-TPO (thyroid peroxidase antibody) concentrations serum samples were performed by laboratory staff at the University Hospital of Northern Norway. The analyses are routine analyses used in the clinic for diagnostic purposes except for T_3_, T_4_, and thyroxine-binding capacity. Analytical methods, instrumentation, analytical variation, quality controls, and method specific reference ranges have been reported by Berg et al. (2015). The laboratory is certified according to ISO 151810 (Norwegian Accreditation 2014). The Norwegian National Unit for Newborn Screening at Oslo University Hospital tested the newborn blood for TSH concentrations. Blood spots were collected on an S&S or Whatman 903 filter paper and analysed with Autodelfia neonatal TSH kits (PerkinElmer).

## Statistical Analyses

Statistical analyses were performed using SPSS statistic software, version 22 (IBM SPSS Inc., Chicago, IL, USA) and R (version 3.1.1; R Project for Statistical Computing). A statistical significance threshold of *p* < 0.05 was used. Only POPs with detection frequencies > 80% were evaluated in statistical models, and concentrations below LODs were replaced by the LOD divided by the square root of 2. All POP concentrations, TSH, and THs were log-normally distributed (Shapiro–Wilk tests) and therefore log_10_-transformed in the statistical analyses. Spearman’s ρ values were calculated for correlations. Statistical analyses were performed including POPs as ng/mL concentrations and repeated using mmol/L concentrations. Initially, partial least square (PLS) regressions were used to evaluate the impact of all POPs and potential covariates simultaneously on maternal serum concentrations of TSH and THs. Separate PLS models were performed with log_10_-transformed and standardized (*z*-scores) variables. For data reduction and to increase the model predictive ability, only variables with variable importance to projection (VIP) values > 0.4 were included in the final model. For highly correlated covariates sensitive to pregnancy-related changes, principal components analysis (PCA) was performed. The score of each woman on the first principal component was included as a common pregnancy vector in multiple linear regression models to avoid collinearity issues while adjusting for these factors. To minimize the number of contaminants to be included in linear regression models, hierarchical clustering analysis of POPs based on correlations (method: complete linkage) was performed, and groupings according to clusters were subsequently performed by simple addition of POP concentrations. Finally, contaminants (individual, grouped or summed, as ng/mL or mmol/L, assessed as quartiles) and covariates were included in multiple linear regression models to report the strength of associations between POPs with TSH and THs. Separate models were built for five dependent variables—TSH, T_3_, T_4_, FT_3_, and FT_4_—where the number of subjects varied between models (*n* = 360–370) according to complete information sets. Diagnostic plots of the residuals and potential influential points were evaluated. Possible confounders were controlled by stratification on variables correlated to both PFOS and THs (results not shown).

## Results

### Population Characteristics

Demographic characteristics of the pregnant women are summarized in Table 1, which shows the variables used as covariates in final statistical models. Characteristics for other variables evaluated as covariates (including iodine status) were decribed in Berg et al. (2015). Demographic characteristics of the newborns are presented in Table 2, which includes available clinical data. The included subjects are representative for pregnant women from the geographical region. The demographic characteristics of the MISA study population and comparison to the general Norwegian pregnant population are reported in detail by Veyhe et al. (2012).

**Table 1:** Maternal concentrations*^a^* of THs, TH-BPs, thyroxine-binding capacity, and maternal characteristics (*n* = 370).

Variable	Median (range)	Mean ± SD	Study population reference range^*b*^
TSH (mlU/L)	1.55 (0.06, 10.2)	1.76 ± 1.04	0.44, 4.48
T_3_ (nmol/L)	2.71 (1.47, 4.75)	2.75 ± 0.46	1.97, 3.73
T_4_ (nmol/L)	145 (92.00, 215)	146 ± 21.1	111, 190
FT_3_ (pmol/L)	4.59 (2.99, 7.08)	4.62 ± 0.53	3.66, 5.79
FT_4_ (pmol/L)	13.0 (9.00, 20.0)	13.4 ± 1.62	10.0, 17.0
Thyroxine-binding capacity^*c*^	1.26 (0.84, 1.50)	1.26 ± 0.09	1.07, 1.43
TBG (mg/L)	36.7 (23.2, 69.6)	37.2 ± 6.74	26.2, 53.3
TTR (g/L)	0.19 (0.09, 0.27)	0.19 ± 0.03	0.15, 0.25
Albumin (g/L)	40.0 (33.9, 47.4)	40.2 ± 2.42	36.0, 46.0
Total lipid (mg/dL)	672 (344, 1,072)	672 ± 126	442, 943
Age	31 (18, 43)	31 ± 5.0
Parity	1 (0, 4)	1± 1.0
Prepregnancy body mass index	23 (17, 40)	24.0 ± 4.3
Body mass index, second trimester	25 (18, 43)	26.0 ± 4.4
Gestational week at blood sampling	18 (10, 34)	18.0 ± 3.4
Physical activity (prepregnancy)^*d*^	6.3 (1, 10)	6.0 ± 1.7
Pregnancy vector^*e*^	–0.01 (–2.5, 3.2)	–0.03 ± 0.9
^***a***^Anti-TPO positive women (*n* = 22) are included in medians. ^***b***^Study population reference range is defined as the 2.5th percentile (lower range) and 97.5th percentile (upper range) for this study population. ^***c***^Unit in TBI = thyroxine-binding index. ^***d***^Reported degree of physical activity on a 1–10 point scale between very seldom to very often. ^***e***^Common vector for pregnancy-related variables, the vector scores includes thyroxine-binding capacity, TBG, TTR, albumin, total lipids, and gestational week. See “Results” for details.			

**Table 2:** Infant TSH concentrations, infant characteristics and study population specific reference range (*n* = 370).

Characteristic	Boys/girls	Median (range)	AM^*a*^ (SD)	Study population reference range^*b*^
Sex	196/174
TSH (mlU/L)		1.20 (0.07, 7.50)	1.32 (0.94)	0.13, 3.90
Gestational length (days)		282 (212, 299)	280 (10.5)
Age at sampling (hr)		72 (48, 364)	74 (23.7)
Birth weight (g)		3,595 (1,330, 4,930)	3,626 (505)
Head circumference (cm)		36 (27, 40)	35.6 (1.46)
Length (cm)		50 (41, 57)	50.3 (2.10)
^***a***^Arithmetic mean. ^***b***^Study population reference range is defined as the 2.5th percentile (lower range) and 97.5th percentile (upper range) for this infant population.				

### Contaminant Concentrations and their Correlations

Seven PFASs were detected in more than 80% of blood samples and were included in the statistical analyses; PFOS (median of 8.03 ng/mL) was the dominating compound, followed by perfluorooctanoate (PFOA, 1.53 ng/mL), perfluorononanoate (PFNA, 0.56 ng/mL), perfluorohexane sulfonate (PFHxS, 0.44 ng/mL), perfluoroundecanoate (PFUnDA, 0.26 ng/mL), perfluorodecanoate (PFDA, 0.23 ng/mL), and perfluoroheptane sulfonate (PFHpS, 0.10 ng/mL). PFAS concentrations and their predictors are described in detail elsewhere (Berg et al. 2014).

Eight polychlorinated biphenyls (PCBs) and four pesticides were detected in > 80% of blood samples and included in the statistical analyses (see Table S1). The highest median wet-weight concentrations were found for *p,p´*-dichlorodiphenyldichloroethylene (*p,p´*-DDE, 0.24 ng/g) followed by PCB-153 (0.16 ng/g), PCB-180 (0.11 ng/g), PCB-138 (0.09 ng/g), hexachlorobenzene (HCB, 0.06 ng/g), PCB-170 (0.04 ng/g), PCB-187 (0.03 ng/g), PCB-118 (0.03 ng/g), PCB-163 (0.02 ng/g), *trans*-nonachlor (0.02 ng/g), PCB-99 (0.01 ng/g), and *cis*-nonachlor (0.004 ng/g). OC concentrations in the entire study population and predictors are described in detail by Veyhe et al. (2015).

The OCs intragroup correlations were higher within the OCs (*r* = 0.54–0.95) compared with the PFASs (*r* = 0.19–0.75) (see Table S2). In the hierarchical clustering analysis, the PCBs were separated in two separate groups and *cis*- and *trans*-nonachlors into one group (see Figure S1). The correlations between the OCs and the PFASs were low and ranged from *r* = 0.13 to 0.50 where the longest chained PFASs were more correlated to the OCs than were the shorter chained compounds.

### Concentrations of Maternal TSH, THs, and TH-BPs and Infant TSH

Maternal concentrations of TSH, THs, and TH-BPs were within nonpregnant reference ranges (Table 1) and time of blood sampling during the day did not influence the variance in concentrations between participants. Twenty-two women had thyroid peroxidase antibodies > 34 IU/L and were categorized as anti-TPO positive according to the manufacturer. The anti-TPO positive women were included in all analyses, tables, and figures because results were unchanged if excluding them. Concentrations of infant TSH are presented in Table 2. The ranges of infant TSH levels were within what is considered a normal reference range (Kapelari et al. 2008). Four infants could be classified with subclinical hypothyroidism as characterized by TSH concentrations > 5 mlU/L (Kaplowitz 2010).

### Maternal TH Concentrations and Associations with POP Concentrations

**Figure 1:**
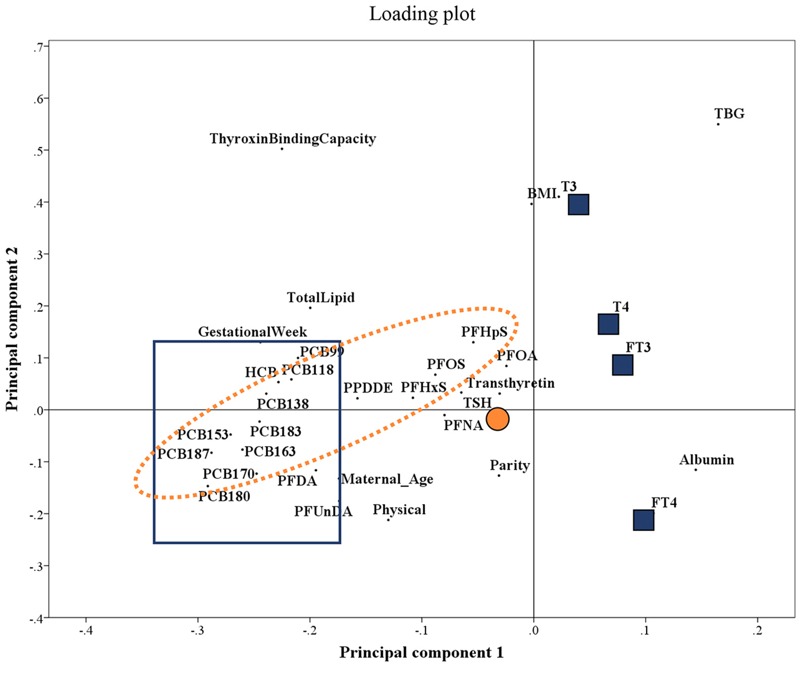
Partial least squares loading plot for TSH and TH concentrations. The plot describes the linear relationship between the independent variables (contaminants and covariates) and the dependent variables (TSH and THs) and how the variables load onto the principal components. POPs circled with a dashed orange line were positively associated with TSH concentrations (marked with a solid orange circle). POPs boxed with a blue solid line were negatively associated with concentrations of T_3_, FT_3_, T_4_, and FT_4_ (marked with blue squares).

PLS regression indicated positive associations between maternal concentrations of TSH and most PFASs and OCs (Figure 1). Further, parity and thyroxine-binding capacity were important covariates for TSH concentrations. The PLS regression also demonstrated an inverse relationship of several OCs, PFDA, and PFUnDA with the THs (Figure 1). Important covariates for these THs were variables related to the course of pregnancy: lipids, albumin, TBG, TTR, thyroxine-binding capacity, and gestational week and these were highly correlated. In the PCA, 50% of the variation in these variables was explained by the first principal component (PC) alone. This PC demonstrated the same association with the THs in the PLS regression plot as the individual variables (results not shown), but to avoid multicollinearity including all the individual variables in multiple regression models, individual PC scores were included as a “common pregnancy-related vector.”

Individual and grouped POPs were included in multiple linear regression models based on the hierarchical cluster analysis (details about the grouped compounds are reported in the Supplemental Material, Section A1, “Summed contaminant groups”) and demonstrated that maternal TSH concentrations were positively associated with PFOS, PCB groups, and the nonachlor group (see Table S3). Further, there were negative associations among *a*) T_3_ and PCB groups, HCB, the nonachlor group and PFDA; *b*) T_4_ and PCB groups, HCB, and the nonachlor group; *c*) FT_4_ and PCB groups; and *d*) FT_3_ and PFUnDA. However, except for associations between PDFA and PFUnDA with T_3_ and FT_3_ (Table 3), associations between the individual and grouped contaminants and THs or TSH were no longer significant when including the other contaminants as covariates interchangeably. Finally, including all the contaminants as summed OCs (sumOCs), summed PFASs (sumPFASs), and summed OCs and PFASs (sumPOPs) in regression models (Table 3) demonstrated a positive association between sumPOPs and TSH, whereas sumOCs were inversely associated with T_3_, T_4_ and FT_4_.

**Table 3:** Associations*^a^* between serum concentrations of POPs with TSH and TH concentrations in pregnant women.

Predictors	*n*	TSH (mlU/L)^*b*^	T_3_ (nmol/L)^*c*^	T_4_ (nmol/L)^*d*^	FT_4_ (pmol/L)^*e*^	FT_3_ (pmol/L)^*e*^
Model 1: SumOCs^*f*^
Quartile 1: 0.18–0.63	90	—	Reference	Reference	Reference	—
Quartile 2: 0.64–0.83	90	—	–0.01 (–0.03, 0.01)	0.001 (–0.02, 0.02)	0.001 (–0.01, 0.02)	—
Quartile 3: 0.84–1.12	91	—	–0.01 (–0.03, 0.01)	–0.004 (–0.02, 0.02)	–0.01 (–0.02, 0.01)	—
Quartile 4: 1.13–4.65	90	—	–0.03 (–0.06, –0.01)*	–0.02 (–0.04, –0.003)*	–0.02 (–0.03, –0.001)*	—
Model 2: SumPFASs^*g*^
Quartile 1: 1.01–8.32	90	—	—	—	—	—
Quartile 2: 8.33–11.7	90	—	—	—	—	—
Quartile 3: 11.8–15.2	91	—	—	—	—	—
Quartile 4: 15.3–45.4	90	—	—	—	—	—
Model 3: SumPOPs^*h*^
Quartile 1: 1.77–9.00	90	Reference	—	—	—	—
Quartile 2: 9.01–12.5	90	0.04 (–0.03, 0.11)	—	—	—	—
Quartile 3: 12.6–16.5	91	0.06 (–0.02, 0.14)	—	—	—	—
Quartile 4: 16.6–46.7	90	0.08 (0.001, 0.16)*	—	—	—	—
Model 4: PFDA
Quartile 1: 0.05–0.17	90	—	Reference	—	—	—
Quartile 2: 0.18–2.3	90	—	–0.01 (–0.030. 0.007)	—	—	—
Quartile 3: 0.24–0.31	91	—	–0.01 (–0.032, 0.005)	—	—	—
Quartile 4: 0.32–2.34	90	—	–0.02 (–0.044, –0.005)*	—	—	—
Model 5: PFUnDA
Quartile 1: 0.02–0.16	90	—	—	—	—	Reference
Quartile 2: 0.17–0.26	90	—	—	—	—	–0.01 (–0.024, 0.004)
Quartile 3: 0.27–0.38	91	—	—	—	—	–0.01 (–0.024, 0.004)
Quartile 4: 0.39–1.46	90	—	—	—	—	–0.02 (–0.033, –0.003)*
^***a***^Regression coefficient β, i.e. change in concentrations (100% × β) across quartiles with the lowest quartile as reference group. ^***b***^The model is adjusted for parity, T-uptake (thyroxine-binding capacity), in addition to sumPFASs and sumOCs in TSH models 1 and 2, respectively. ^***c***^The model is adjusted for pregnancy-related change vector, parity, age, BMI, physical activity, and sumPFASs. ^***d***^ The model is adjusted for pregnancy-related change vector, age, physical activity, and sumPFASs. ^***e***^The model is adjusted for pregnancy-related change vector, age, body mass index, and sumPOPs. ^***f***^Includes PCBs 99,118,138,153,163,170,180, and 187,* p,p*´-DDE, HCB, and *cis*- and *trans*-nonachlor. ^***g***^Includes PFHpS, PFHxS, PFOA, PFOS, PFNA, PFDA, and PFUnDA. ^***h***^Includes SumOCs and SumPFASs. *p < 0.05, calculated for the change in concentrations compared with the reference quartile.						

The results from PLS regression, hierarchical cluster analysis, and linear regressions were consistent regardless of including POP concentrations as either ng/mL or mmol/L (results not shown). Further, the regression coefficients for associations between OCs and THs were the same regardless of including wet-weight or lipid-adjusted OC concentrations in the models. However, the associations between OCs and T_3_, T_4_, and FT_4_ were stronger for wet-weight than for lipid-adjusted concentrations. Parity and age were correlated with several POPs, TSH, and THs, and repeating the analyses when stratifying on parity and age groups demonstrated the same associations between POPs and TSH and THs as the full models (results not shown). Because residual variance related to pregnancy-related changes could affect blood lipids, the analyses were repeated in a subset of women in gestational week 20 only (*n* = 94). In these analyses, including the OCs as wet-weight or lipid-adjusted concentrations resulted in the same regression coefficients (results not shown).

### Associations Between Levels of Maternal TH and Infant TSH

The infant TSH concentrations 3 days after birth were positively associated with maternal TSH concentrations in second trimester and inversely associated with maternal FT_4_ concentrations 3 days postpartum (see Table S4). The children classified with subclinical hypothyroidism had mothers within the highest TSH and PFOS quartile. No associations between maternal POP concentrations with infant TSH concentrations or maternal POP and TH concentrations with birth outcomes were observed.

## Discussion

### Main Findings

To our knowledge, this is the first multipollutant study investigating thyroid disrupting effects of both OCs and PFASs, including infant TSH concentrations and as many as 10 thyroid hormone parameters in pregnant women. Background exposures of POPs influenced maternal concentrations of TSH and THs, whereas infant TSH concentrations were associated with maternal concentrations of TSH and FT_4_. The study results contribute to a comprehensive understanding of the maternal thyroid hormone homeostasis in relation to current composite POP concentrations.

### Associations Between Concentrations of POPs and Maternal TSH and THs

Several individual PFASs and OCs were associated with maternal TSH and THs, but only PFDA and PFUnDA were significantly associated with THs after adjusting for other POPs. We could therefore not separate the importance of most compounds because it is likely that they shared variance in TSH and TH concentrations in the linear regression models. Accordingly, including individual or grouped variables of PFASs, OCs, and POPs in linear regression models demonstrated the same overall associations with TSH and THs that is demonstrated by comparing Table 3 and Table S3. However, the associations between individual POPs, mainly PFOS and PCBs with TSH, were stronger compared with those of sumPOPs with TSH, and this may be explained by higher random variation in the latter regression equations. Concentrations of the PFASs were 10-fold higher than the OCs and were highly correlated with the sumPOP variable (*r* = 0.98) (Table 3). This likely explains why the results of regressions including sumPOPs resembled those for sumPFASs.

Recent literature reports relevant mechanisms of thyroid disruption by POPs in general to be *a*) disturbance of the overall activity of the thyroid gland by interference with the TH receptors; *b*) stimulation or inhibition of enzyme functions that mediate iodine uptake of the thyroid gland in the synthesis of T_3_ and T_4_; and *c*) competitive displacement of THs on their binding proteins (Boas et al. 2012). Because the PFASs and OCs seem to be differentially associated with concentrations of THs, this could indicate different modes of action with regard to the effect of PFASs and OCs on the maternal thyroid homeostasis, but any conclusion is not feasible based solely on statistical associations. It has been hypothesized that PFOS can alter the thyroid hormone levels by competitive binding to TH-BPs, and does not affect the regulatory functions of the thyroid hormone system itself as is demonstrated for the OCs (Chang et al. 2008; Lau et al. 2007). However, the associations between OCs and T_3_, T_4_, and FT_4_ may also reflect a compensatory feedback mechanism of elevated TSH levels due to PFASs exposure, or the opposite, where TSH levels are elevated as a response to disrepancies in TH levels due to disruption by OCs.

### The Influence of Maternal POP Exposures on Infant Thyroid Function

We did not observe associations between maternal POP concentrations and concentrations of infant TSH. However, interpretation of TSH concentrations 3 days after birth in regard to dysregulation of infant TH homeostasis may be too early to indicate thyroid impairment, and divergences in TH levels due to maternal POP exposures could develop throughout childhood. Indeed, associations between PCB concentrations in breast milk and TH levels in 1-year-old children have been demonstrated (Nagayama et al. 1998), and prenatal exposure to PCBs and dioxin was reported to be associated with subtle cognitive and motor developmental delays in children at school age (Vreugdenhil et al. 2002). Still, in a different study PCBs 99, 138, 153, 180, 183, 187, 194, and 199 were positively associated with neonatal TSH concentrations measured 3 days after birth (Chevrier et al. 2007), and concentrations of T_3_ in 3-week-old infants were inversely associated with low-chlorinated PCBs (Darnerud et al. 2010). The discrepancies between these studies and the present study may be explained by different sampling periods between the studies, where women in the two previous studies (Chevrier et al. 2007; Darnerud et al. 2010) were sampled in the years 1996–2000 and had 3-fold higher POP concentration compared with the present study population.

### Multipollutant Assessments of POPs

Hierarchical clustering demonstrated distinct clusters dividing the PFASs and OCs into separate groups. This is in line with their physicochemical properties, but may also partly reflect the difference in their concentrations and temporal trends (Nøst et al. 2013, 2014). Stronger correlations within the OCs could indicate more homogeneous exposure to the different OCs (Lohmann et al. 2007) compared with the PFASs, whereas stronger correlations between the longest chained PFASs and the OCs may reflect similar recent exposure routes and persistence for these compounds. Dallaire et al. (2009) reported comparable correlations between PFOS and OCs (*r* = 0.36–0.55 vs. 0.25–0.45 in the present study) and between the different OCs (*r* = 0.69–0.98 vs. 0.54–0.95), even though concentrations were higher and the sampling was performed in 2004 in that study.

Assessing the impairment of physiological processes by contaminants is complicated by the complex correlation of exposures because one specific POP that is associated in a given study may partly or largely reflect the influence of other POPs rather than the impact of that POP itself. Hence, we cannot exclude that the observed associations between POPs and concentrations of TSH and THs are related to other contaminants (e.g., brominated flame retardants, bisphenols, and phthalates) not included in the statistical analyses. When assessing individual contaminants, we demonstrated significant associations between PFOS and TSH. Accordingly, PFOS was positively associated with TSH in pregnant women in Norway sampled in 1999–2008 (Wang et al. 2013). Further, we demonstrated that PCBs, HCB, and nonachlors were inversely associated with T_3_, T_4_, and FT_4_. These results are in accordance with the work of Chevrier et al. (2008), who reported inverse associations between OCs (e.g., PCBs and HCB) and T_4_ and FT_4_ in pregnant women sampled in 1999–2000, and of Takser et al. (2005), who demonstrated inverse associations between concentrations of T_3_ and PCBs 138, 153, and 180. However, in the present study, the only associations between single contaminants and THs that remained significant when including the other contaminants as covariates were the associations of PFDA and PFUnDA with T_3_ and FT_3_, respectively (Table 3). In other studies, authors have also been unable to pinpoint the individual effect of OCs on TH status when controlling for other OCs because of the high intercorrelations between compounds (Chevrier et al. 2008; Dallaire et al. 2009; Takser et al. 2005). The latter studies therefore also applied summed OCs in their statistical models. The summing of POP groups in the present study were based on intra- and intergroup correlations of the POPs, so we did not consider similar molecular mechanisms in the summing strategy. Finally, summing the concentrations of POPs assumes equal potencies and no synergistic effects, which may mask effects of individual compounds.

### Clinical Relevance

The 95% confidence interval for the maternal and infant thyroid parameters varied within what is considered normal reference ranges for healthy nonpregnant and infant populations (Norwegian Association of Medical Biochemistry 2015), so the clinical relevance of the observations is not obvious. Still, infants born to mothers within the highest TSH quartile had 10% higher mean concentrations of TSH compared with children born to mothers in the lowest TSH quartile. This indicates an influence of maternal thyroid function on the infant TSH levels and could be a transferred effect of POP influence on the maternal thyroid homeostasis. Any disruption of maternal thyroid homeostasis during pregnancy and impairment in infant TH levels is indicated to affect infant development according to Morreale de Escobar et al. (2000, 2004). Maternal hypothyroidism with high TSH and low FT_4_ levels increases the risk of premature birth, preeclampsia, low birth weight, and impaired neuropsychological development in childhood (Burman 2009; Davis et al. 1988), whereas a decrease in maternal FT_4_ due to mild iodine deficiency may affect cognitive function of the offspring (Abalovich et al. 2007). Although a pregnant woman’s hypothyroidism is subclinical (mild and asymptomatic), it can still influence fetal neurodevelopment (Berbel et al. 2009).

### Strengths and Limitations

Due to the complexity of the thyroid system, especially during pregnancy, assessment of potential thyroid impairment cannot be interpreted solely from the individual thyroid parameters. Therefore, we included all major components in the maternal thyroid homeostasis. To account for the adaptations in metabolic processes in pregnant women, we performed a thorough assessment of pregnancy-related covariates, and several influenced the variation in TSH and TH concentrations. As variables (e.g., thyroxine-binding capacity, TBG, TTR, lipids, and gestational week) influencing TH concentrations were highly correlated, they were included into a common pregnancy vector, thereby enabling us to adjust for all of them in multiple regressions instead of selecting individual ones. If these latter variables were not adjusted for in the statistical models (individually or included in the vector), many more PFASs and OCs were significantly associated with THs. Still, many of these covariates are not regarded in the majority of studies on POPs influence on THs in pregnant women.

When thyroid function in pregnant women is evaluated, measurement of FT_4_ is recommended because free hormone reflects the physiological effects on thyroid hormones better than total hormone concentrations due to the pregnancy-related increases in TH-BPs (Fitzpatrick and Russell 2010). Still, these changes could also mask an actual decrease in levels of T_3_, T_4_, FT_3_, and FT_4_. The natural interference on THs by physiological changes during pregnancy could be reduced if all the pregnant women were sampled at the exact same gestational week. Because this was not possible in the present study, we included the TH-BPs, thyroxine-binding capacity (which reflects elevated levels of all the TH-BPs), lipids, and gestational week as a proxy for the pregnancy-related alterations in blood in statistical models. This was supported by repeated analyses of subsets of women in gestational week 18 and 20 where the associations from full models were confirmed.

The wet-weight concentrations of OCs were used in the regression models to be comparable with the PFAS wet-weight concentrations. Due to the mutual dependency between OCs and THs with lipids (results not shown), we chose to use the wet weight concentrations in the regression models for THs. Further, total lipids were adjusted for in models included in the pregnancy vector; an additional adjustment for lipids of the OCs was not performed because it would probably have led to overadjustments. This was confirmed in analyses performed on women in gestational week 20, where the effect of pregnancy-related variables was assumed to be similar, and where using either wet-weight or lipid-adjusted concentrations gave the same regression coefficients.

An important problem in multiple statistical comparisons is that the probability of wrongly concluding that there is at least one statistically significant effect across a set of tests increases with each additional test (Gelman et al. 2012). To minimize multiple comparisons, regression models in the present study were built on the overall associations from one PLS-regression model and not from several simple linear regression models.

## Conclusions

The present study suggests that background exposure of POPs can alter thyroid hormone homeostasis in pregnant women, subsequently affecting the infant thyroid system. Our results highlight the challenges of assessing effects on thyroid function, especially during pregnancy, due to the complexity of contaminant mixtures and the thyroid system. However, regarding the critical role of maternal thyroid hormones in fetal development, associations between maternal thyroid homeostasis with individual, grouped, or summed POPs are of great public health importance.

## Supplemental Material

(253 KB) PDF
