# Supplementary material for: Persistent Organic Pollutants and the Association with Maternal and Infant Thyroid Homeostasis: A Multipollutant Assessment
Source: Environ Health Perspect. 2016 May 24;125(1):127–33. doi: 10.1289/EHP152 (PMC5226691; doi:10.1289/EHP152)
Supplement: (253 KB) PDF [file EHP152.s001.acco.pdf]

Environ Health Perspect
DOI: 10.1289/EHP152

Note to readers with disabilities: *EHP* strives to ensure that all journal content is accessible to all readers. However, some figures and Supplemental Material published in *EHP* articles may not conform to 508 standards due to the complexity of the information being presented. If you need assistance accessing journal content, please contact ehp508@niehs.nih.gov. Our staff will work with you to assess and meet your accessibility needs within 3 working days.

Supplemental Material

Persistent Organic Pollutants and the Association with Maternal and Infant Thyroid Homeostasis: A Multipollutant Assessment

Vivian Berg, Therese Haugdahl Nøst, Rolf Dagfinn Pettersen, Solrunn Hansen, Anna-Sofia Veyhe, Rolf Jorde, Jon Øyvind Odland, and Torkjel Manning Sandanger

Table of Contents

Table S1: Wet-weight and lipid-adjusted concentrations of OCs in serum from a subset of pregnant women in the MISA study (n= 370)

Table S2: Spearmans rank correlation coeficients

Figure S1. Hierarchical clustering of 19 POPs based on concentrations in 391 serum samples. The figure depicts the hierarchical structure obtained from the correlation between compounds (method: complete linkage). The vertical black line represents the manually selected cut-off for the number of clusters marked with boxes.

A1. Summed contaminant groups

Table S3: Linear regression coefficients[a] (95% CI) for associations between maternal concentrations of POPs (ng/mL) and TSH and THs.

Table S4: Linear regression coefficients[a] (95% CI) for associations between concentrations of maternal TSH and FT4 with infant concentrations of TSH.

Table S1: Wet-weight and lipid-adjusted concentrations of OCs in serum from a subset of pregnant women in the MISA study (n= 370)

	**Wet-weight (ng/g serum)**			**Lipid-adjusted (ng/g lipids)**		
**Compound[a]**	**Median**	**AM[b]**	**Range**	**Median**	**AM[b]**	**Range**
PCB 99	0.014	0.016	0.004, 0.075	2.12	2.41	0.44, 10.9
PCB 118	0.026	0.031	0.007, 0.228	3.99	4.63	0.94, 38.3
PCB 138	0.095	0.109	0.016, 0.670	14.2	16.3	2.10, 91.3
PCB 153	0.160	0.183	0.026, 1.247	23.8	27.5	3.39, 170
PCB 163	0.022	0.027	0.006, 0.181	3.39	3.99	0.72, 24.6
PCB 170	0.042	0.049	0.006, 0.434	6.43	7.40	0.75, 59.2
PCB 180	0.105	0.124	0.017, 1.163	15.9	18.7	3.01, 159
PCB 187	0.029	0.033	0.004, 0.168	4.30	4.93	0.81, 26.6
p,p´-DDE	0.243	0.292	0.056, 2.445	36.3	43.9	7.49, 344
HCB	0.062	0.068	0.021, 0.317	9.35	10.2	2.81, 53.3
t-Nonachlor	0.018	0.021	0.004, 0.129	2.72	3.18	0.59, 13.8
c-Nonachlor	0.004	0.005	0.001, 0.033	0.63	0.76	0.08, 3.57

[a]PCB, polychlorinated biphenyl; *p,p*'-DDE, dichlorodiphenyldichloroethylene; HCB, hexachlorobenzene. Values are presented for compounds with detection frequencies >80%; levels below the LOD were set to LOD/√2.

[b]AM = Arithmetic mean

Table S2: Spearmans rank correlation coeficients

	PCB 99	PCB 118	PCB 138	PCB 163	PCB 153	PCB 170	PCB 180	PCB 187	*p,p*-DDE	HCB	*t*-Nonachlor	*c*-Nonachlor	PFHxS	PFOA	PFHpS	PFOS	PFNA	PFDA
PCB118	.76																	
PCB138	.89	0.79																
PCB163	.66	0.66	0.75															
PCB153	.84	0.79	0.95	0.83														
PCB170	.65	0.62	0.78	0.82	0.87													
PCB180	0.67	0.69	0.83	0.82	0.93	0.91												
PCB187	0.78	0.76	0.89	0.81	0.95	0.86	0.93											
p,p'-DDE	0.77	0.68	0.8	0.6	0.76	0.61	0.62	0.7										
HCB	0.77	0.73	0.77	0.7	0.77	0.65	0.65	0.7	0.64									
t-Nonachlor	0.77	0.75	0.79	0.75	0.82	0.71	0.76	0.82	0.62	0.77								
c-Nonachlor	0.69	0.67	0.7	0.66	0.71	0.61	0.65	0.71	0.54	0.66	0.9							
PFHxS	0.35	0.39	0.36	0.32	0.32	0.27	0.24	0.25	0.27	0.39	0.36	0.34						
PFOA	0.25	0.28	0.29	0.28	0.26	0.23	0.17	0.18	0.26	0.34	0.19	0.13	0.53					
PFHpS	0.31	0.33	0.29	0.23	0.25	0.16	0.17	0.2	0.22	0.33	0.31	0.3	0.5	0.46				
PFOS	0.4	0.42	0.37	0.36	0.35	0.27	0.25	0.27	0.29	0.45	0.41	0.39	0.63	0.65	0.68			
PFNA	0.32	0.43	0.37	0.39	0.38	0.34	0.34	0.34	0.29	0.42	0.37	0.33	0.49	0.64	0.45	0.61		
PFDA	0.35	0.43	0.39	0.37	0.41	0.38	0.39	0.39	0.3	0.39	0.41	0.41	0.45	0.47	0.41	0.58	0.75	
PFUnDA	0.39	0.45	0.42	0.4	0.45	0.39	0.44	0.46	0.32	0.4	0.49	0.5	0.34	0.19	0.28	0.44	0.55	0.75

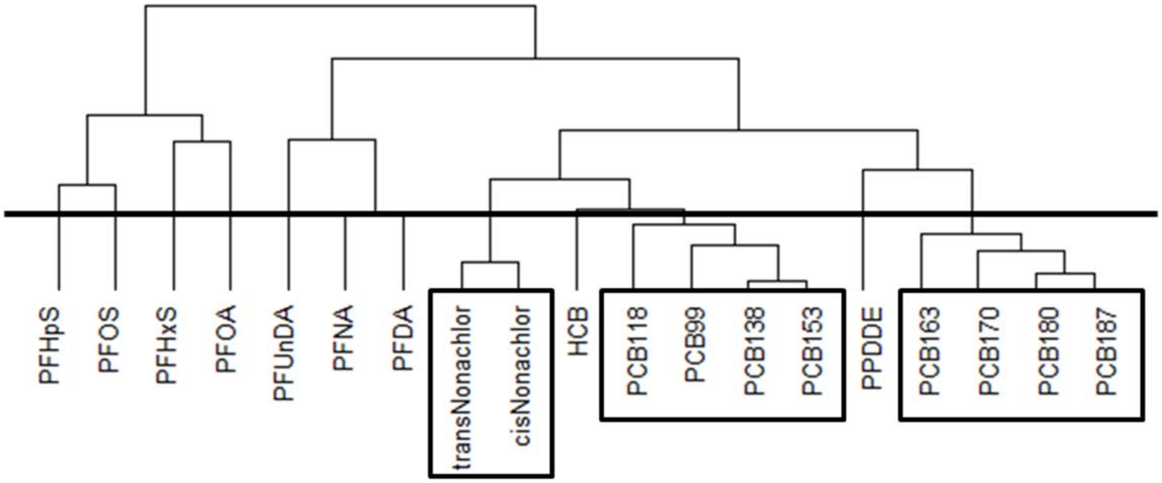

Figure S1. Hierarchical clustering of 19 POPs based on concentrations in 391 serum samples. The figure depicts the hierarchical structure obtained from the correlation between compounds (method: complete linkage). The vertical black line represents the manually selected cut-off for the number of clusters marked with boxes.

A1. Summed contaminant groups

Based on the hierarchical clustering analysis, groups were established by addition of concentrations; i) Nonachlor (*cis*-nonachlor and *trans*-nonachlor); ii) PCB 99-153 (PCB 99, 118, 138, 153) and v) PCB 163-187 (PCB 138, 153, 163, 170, 180, 187). The remaining compounds were kept as single compounds.

Table S3: Linear regression coefficients[a] (95% CI) for associations between maternal concentrations of POPs (ng/mL) and TSH and THs.

	n	Model 1: T3[b]	R^2	Model 2: T4[c]	R^2	Model 4: TSH[d]	R^2	Model 3: FT4[e]	R^2	Model 4: FT3[e]	R^2
**PCB 99-153[f]**											
Quartile 1	91	Reference		Reference		Reference		-		-	
Quartile 2	91	-0.002 (-0.02, 0.016)		0.003 (-0.015, 0.02)		0.04 (-0.03, 0.11)		-		-	
Quartile 3	91	-0.02 (-0.04, 0.001)		-0.01 (-0.03, 0.01)		0.03 (-0.04, 0.10)		-		-	
Quartile 4	91	-0.03 (-0.05, -0.01)*	30	-0.02 (-0.04, -0.01)*	7	0.08 (0.01, 0.16)	8	-	-	-	-
**HCB**											
Quartile 1	92	Reference		Reference		-		-		-	
Quartile 2	92	-0.01 (-0.03, 0.007)		-0.004 (-0.02, 0.01)		-		-		-	
Quartile 3	93	-0.02 (-0.04, -0,003)*		-0.006 (-0.02, 0.01)		-		-		-	
Quartile 4	92	-0.02 (-0.05, -0,01)**	31	-0.03 (-0.05, -0,01)*	8	-	-	-	-	-	-
**Nonachlors[g]**											
Quartile 1	92	Reference		Reference		Reference		-		-	
Quartile 2	92	-0.01 (-0,03, 0.008)		-0.002 (-0.02, 0,02)		0.09 (0.024, 0.163)**		-		-	
Quartile 3	93	-0.02 (-0.04, 0.003)		-0.01 (-0.03, 0.01)		0.07 (0.002, 0.145)*		-		-	
Quartile 4	92	-0.03 (-0.05, -0 01)*	30	-0.02 (-0.04, -0.01)*	7	0.01 (0.010, 0.159)*	9	-	-	-	-
**PCB 163-187[h]**											
Quartile 1	92	Reference		Reference		Reference		Reference		-	
Quartile 2	92	-0.02 (-0.03, -0.002)*		-0.01 (-0.02,0.01)		0.04 (-0.03, 0.11)		-0.002 (-0.016, 0.012)		-	
Quartile 3	93	-0.02 (-0.04, -0.001)*		-0.01 (-0.02,0.01)		0.04 (-0.03, 0.11)		0.001 (-0.019, 0.009)		-	
Quartile 4	92	-0.04 (-0.06, -0,02)**	31	-0.03 (-0.05, -0.06)*	8	0.10 (0.03, 0.17)**	7	-0.02 (-0.030, -0.001)*	26	-	-
**PFOS**											
Quartile 1	94	-		-		Reference		-		-	
Quartile 2	87	-		-		0.04 (-0.031, 0.114)		-		-	
Quartile 3	95	-		-		0.08 (0.006, 0.154)*		-		-	

Quartile 4	94	-	-	-	-	0.10 (0.016, 0.169)*	8	-	-	-	-
**PFUnDA**											
Quartile 1	91	-		-		-		-		Reference	
Quartile 2	93	-		-		-		-		-0.01 (-0.024, 0.004)	
Quartile 3	93	-		-		-		-		-0.01 (-0.024, 0.004)	
Quartile 4	93	-	-	-	-	-	-	-	-	-0.02 (-0.033, -0.003)*	14
**PFDA**											
Quartile 1	92	Reference		-		-		-		-	
Quartile 2	93	-0.01 (-0.030. 0.007)		-		-		-		-	
Quartile 3	93	-0.01 (-0.032, 0.005)		-		-		-		-	
Quartile 4	92	-0.02 (-0.044, -0.005)*	30	-	-	-	-	-	-	-	-

*p≤0.05, **p<0.01 calculated for the change in concentrations compared to the reference quartile.

[a]Regression coefficient β, i.e. change in concentrations (100% x β) across quartiles with the lowest quartile as reference group.

[b]The model is adjusted for pregnancy related change vector, parity, age, BMI and physical activity

[c]The model is adjusted for pregnancy related change vector, age and physical activity

[d]The model is adjusted for t-uptake and parity

[e]The model is adjusted for pregnancy related change vector, BMI and age

[f]Includes PCB 99,118,138 and 153

[g]Includes *trans*- and *cis*-nonachlor

[h]Includes PCB 163, 170, 180 and 187

R^2 presented as %

Table S4: Linear regression coefficients[a] (95% CI) for associations between concentrations of maternal TSH and FT4 with infant concentrations of TSH.

Predictors	n	Infant TSH mlU/L[b]
**Model 1: Maternal TSH mlU/L[a]**		
Quartile 1: 0.06-1.09	92	Reference
Quartile 2: 1.11-1.55	94	0.03 (-0.063, 0.131)
Quartile 3: 1.56-2.16	90	-0.03 (-0.134, 0.065)
Quartile 4: 2.19-10.17	92	0.10 (0.001, 0.201)*
**Model 2: Maternal FT4 pmol/L**		
Quartile 1: 10-13	104	Reference
Quartile 2: 13.3-14	80	-0.08 (-0.176, 0.007)
Quartile 3: 14-16	103	-0.05 (-0.147, 0.049)
Quartile 4: 16-25	81	-0.11 (-0.204, -0.014)*

*p<0.05, calculated for the change in concentrations compared to the reference quartile.

[a]Regression coefficient β, i.e. change in concentrations (100% x β) across quartiles with the lowest quartile as reference group.

[b]The models are adjusted for maternal total lipid, infant age at sampling, birthweight, gender and gestational length.
